# Neurological Complications After Thoracic Endovascular Repair (TEVAR): A Narrative Review of the Incidence, Mechanisms and Strategies for Prevention and Management

**DOI:** 10.3390/jpm16020077

**Published:** 2026-02-01

**Authors:** Francesca Miceli, Marta Ascione, Rocco Cangiano, Antonio Marzano, Alessia Di Girolamo, Giovanni Gagliardo, Luca di Marzo, Wassim Mansour

**Affiliations:** Vascular and Endovascular Surgery Division, Department of General Surgery and Surgical Specialties, Policlinico Umberto I, “Sapienza” University of Rome, Viale del Policlinico 155, 00161 Rome, Italy; marta.ascione@uniroma1.it (M.A.); rocco.cangiano@uniroma1.it (R.C.); antonio.marzano@uniroma1.it (A.M.); alessia.digirolamo@uniroma1.it (A.D.G.); giovanni.gagliardodicarpinello@uniroma1.it (G.G.); luca.dimarzo@uniroma1.it (L.d.M.); wassim.mansour@uniroma1.it (W.M.)

**Keywords:** TEVAR, spinal cord ischemia, stroke, delirium, aortic disease, neuroprotection, personalized medicine

## Abstract

**Background**: Thoracic endovascular aortic repair (TEVAR) has evolved the management of descending thoracic aortic disease, but neurological complications—particularly spinal cord ischemia (SCI), stroke, and postoperative delirium—remain among the most feared adverse events, adversely affecting survival, quality of life, and functional independence. **Objectives**: The aim of this study was to provide a contemporary narrative synthesis (2000–2025) of the incidence, mechanisms, risk factors, prevention, and management of neurological complications after TEVAR, emphasizing how current evidence supports individualized and risk-adapted strategies for prevention and management. **Methods**: A narrative, non-systematic search (PubMed/MEDLINE, Scopus, Cochrane Library; 2000–2025) was conducted using terms related to TEVAR, SCI, cerebrovascular events, delirium, and cognitive dysfunction. Priority was given to large registries, cohort studies, and systematic reviews in adult TEVAR populations. **Results**: Perioperative stroke occurs in ~2–6% of TEVAR cases, with higher rates in arch/zone 0–2 procedures and when the left subclavian artery (LSA) is covered without revascularization. SCI incidence ranges from ~2–9%, influenced by aortic extent and urgency; Vascular Quality Initiative data report SCI in 3.7% of procedures, with markedly reduced 1-year survival. Major SCI risk factors include extensive thoracic coverage, prior aortic repair, vertebral or hypogastric occlusion, emergency presentation, low perioperative mean arterial pressure, anemia, and chronic kidney disease. Postoperative delirium occurs in ~13% of TEVAR-treated type B dissections and correlates with longer hospitalization and early complications. Emerging nomograms for SCI and delirium enable individualized risk stratification. **Conclusions**: Neurological complications after TEVAR remain clinically significant. Contemporary evidence supports personalized prevention—selective cerebrospinal fluid (CSF) drainage, LSA revascularization, staging, neuromonitoring, and tailored hemodynamic targets—guided by anatomical complexity, comorbidities, collateral network integrity, and prior aortic history. Further research should refine prediction tools, standardize definitions, and evaluate individualized neuroprotective bundles.

## 1. Introduction

Thoracic endovascular aortic repair (TEVAR) has become the “gold standard” treatment for several descending thoracic aortic pathologies—such as complicated type B aortic dissections, penetrating aortic ulcers, traumatic aortic injuries, and many degenerative aneurysms—because it offers a reduction in early morbidity and mortality compared with traditional open thoracic aortic repair [1]. Involvement in perioperative care and device technology has expanded its application and enhanced procedural safety over the past several decades, making it possible to treat high-risk patients and complex aortic anatomies that were previously considered inoperable. Despite these developments, one of the most frightening side effects of TEVAR is still neurological concerns. Postoperative delirium, ischemic stroke, and spinal cord ischemia (SCI) all have the potential to change a patient’s postoperative course. As TEVAR become more widely adopted, it has become evident that these neurological outcomes are not incidental or random but result from complex interactions between the procedure, the patient, and the underlying disease. The anatomy of the thoracic and thoracoabdominal aorta—the extent of stent-graft coverage, the proximity to the left subclavian artery, and the patency of vertebral and hypogastric arteries—plays a critical role in the risk of neurological complications. The degree of arch manipulation, intraoperative hemodynamic conditions and the endovascular device’s design and conformability all influence the risk of embolic or hypoperfusion-related neurological complications. Furthermore, interindividual heterogeneity in neurological susceptibility is significantly influenced by patient-specific variables, such as elderly, frailty, cerebrovascular disorders, chronic renal disease, and pre-existing cognitive impairment. Previous aortic surgery, whether endovascular or open, may decrease collateral perfusion paths, making patients more susceptible to SCI. Furthermore, evidence indicates that the individual risk profile may also be influenced by systemic inflammatory responses, microvascular integrity, and genetic predisposition, even though these factors are still poorly understood [2,3].

It is crucial to assess the variability of neurological problems following TEVAR to create efficient, personalized prevention measures such as neuromonitoring, staged repair strategies, permissive hypertension protocols, left subclavian artery revascularization and selective cerebrospinal fluid drainage. In addition to anatomical and procedural factors, risk stratification should include patient’s comorbidities, functional status, and cognitive reserve, especially in older or frail patients. Pre-operative evaluation should take neurological damage into account since it may have significant effects on postoperative quality of life.

We provide a contemporary non-systematic narrative review (from 2000 to 2025) of the incidence, mechanisms, risk factors, prevention, and management of neurological complications after TEVAR, emphasizing how current evidence supports patient’s risk-adapted strategies for prevention and management.

## 2. Materials and Methods

We performed a narrative non-systematic review of the literature across three major databases: PubMed/MEDLINE, Scopus, and the Cochrane Library. The search covered the period from January 2000 to December 2025. Search terms included combinations of “thoracic endovascular aortic repair” OR “TEVAR”, “spinal cord ischemia” OR “spinal cord injury” OR “paraplegia”, “stroke” OR “cerebrovascular events” OR “cerebrovascular accident”, “delirium” OR “postoperative delirium” OR “cognitive dysfunction” OR “neurocognitive”. Only English-language studies involving human subjects were considered. We focused on original articles, systematic reviews and meta-analyses specifically addressing neurologic complications after TEVAR. We also searched for TEVAR-related neurologic outcomes in large registries (e.g., Vascular Quality Initiative, GREAT registry) [2,3].

Editorials, expert opinions without original data, case reports and studies not reporting neurological outcomes or not specifically focused on TEVAR in adult patients were excluded.

Although we did not apply a formal quality assessment tool such as ROBIS or PRISMA, we critically appraised the clinical relevance, methodological clarity and applicability of each study included in our discussion. Because this is a narrative rather than systematic review, no formal risk-of-bias scoring or quantitative meta-analysis was performed. Instead, we synthesized major themes and highlighted consistent findings across high-quality studies. The aim was not to perform a systematic quantitative analysis, but rather to offer a pragmatic, experience-driven overview of preventive strategies, clinical outcomes and management of TEVAR- related neurological complications.

## 3. Overview of Neurological Complications After TEVAR

Following TEVAR, neurological complications are a major clinical issue. Spinal cord ischemia (SCI) and ischemic stroke continue to be the most common carrying a significant effect on survival and long-term function [4,5]. As reported by Ullery et al. [6], these complications derive from multifactorial factors involving patient-specific anatomy, type of procedural complexity, and perioperative course. In addition to these major events, postoperative delirium and cognitive impairment have become increasingly recognized as significant causes of morbidity, especially in elderly and frail patients [7]. Less frequent manifestations, such as seizures, peripheral nerve injury and cortical blindness secondary to embolic phenomena, complete the neurological spectrum associated with TEVAR [8].

### 3.1. Cerebrovascular Events After TEVAR

Stroke remains one of the most serious neurological complications following TEVAR. Stroke incidence varies across studies depending on procedural technique, with systematic reviews reporting values between 3% and 6% [9,10]. Its pathogenesis is multifactorial. The dominant mechanism is embolization: atherothrombotic material can be dislodged by manipulating guidewires, catheters, and large-bore delivery systems in a highly atherosclerotic aorta, especially at the level of the aortic arch [11]. In addition, hypoperfusion—especially during periods of intraoperative or perioperative hypotension—may impair cerebral perfusion, particularly in patients with pre-existing cerebrovascular disease or compromised collateral flow. A third mechanism occurs when the left subclavian artery (LSA) is covered without revascularization. This compromises posterior circulation and reduces vertebrobasilar supply, particularly in patients with a dominant left vertebral artery or insufficient contralateral flow. Recent clinical evidence reported the significant risk of stroke following TEVAR, particularly when the aortic arch is affected [12]. The analysis of the Society for Vascular Surgery Vascular Quality Initiative (VQI) registry, including patients undergoing TEVAR with proximal landing in zones 0–2, reported an experienced in-hospital stroke rates of 11.1% in zone 0, compared with 5.3% and 4.7% in zones 1 and 2, respectively. These findings demonstrated a more than two-fold increased risk of stroke in arch (zone 0) procedures [13]. Another recent study showed that in 3072 elective TEVARs for degenerative aneurysm, the 30-day stroke rate was 7.1% when performed for arch aneurysms, versus 2.9% when for descending (non-arch) aneurysms (*p* = 0.001). Independent predictors included advanced age (>79 years), functional status, endovascular supra-aortic trunk revascularization, and longer procedural time—highlighting how patient frailty and procedural complexity influence cerebrovascular risk [14]. These findings carry important implications for clinical practice. Preoperative evaluation should include a computed tomography angiography (CTa) of the aortic arch and supra-aortic trunks to assess the presence of heavy calcification, arch anatomy, vertebral artery dominance, and feasibility of cerebral protection strategies (Level of evidence C) [15]. In patients requiring proximal landing zones (especially zone 0), the increased risk documented in recent data argues strongly in favor of hybrid surgical debranching rather than total endovascular arch repair when feasible [16], or at least selective revascularization of the LSA/vertebral inflow to preserve vertebrobasilar circulation [15]. The use of soft-tipped wires, controlled deployment, limiting arch manipulation, and minimizing wire exchanges are all crucial aspects of intraoperative technique [17]. As important is the hemodynamic management, which includes maintaining adequate cerebral perfusion and preventing hypotension, especially in individuals with incomplete Circle of Willis or established carotid disease. Some centers have begun to adopt adjunct neuromonitoring (e.g., cerebral oximetry) to detect early desaturation. Strict neurological surveillance, timely neuroimaging when any impairment is suspected, and optimization of antithrombotic treatment are crucial since many strokes occur in the early post-operative period. In selected patients with vertebrobasilar insufficiency risk, early blood-pressure optimization or even intra-operative hypertension (Level of Evidence C) [15,18] may be considered to support cerebral perfusion.

### 3.2. Spinal Cord Ischemia After TEVAR

#### 3.2.1. Pathophysiology

**Figure 1:**
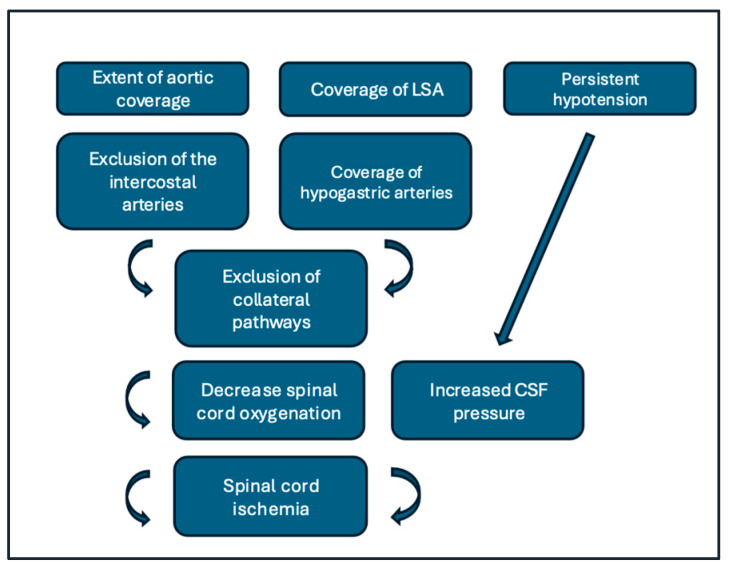
Flow-chart summarizing the pathophysiology of the spinal cord ischemia after TEVAR.

The pathophysiology of SCI following TEVAR relies on a steady equilibrium between arterial inflow, collateral vessels, and perfusion pressure (Figure 1). The spinal cord is supplied by a longitudinal system, mainly by the anterior spinal artery (ASA) and paired posterior spinal arteries, which receive inflow from several segmental vessels arising from the vertebral, intercostal, lumbar, and internal iliac arteries. Griepp and colleagues described the “collateral network concept,” underlining that spinal cord perfusion is not dependent on a single “critical” artery but on a dense network that includes intercostal and lumbar segmental arteries, the vertebral and subclavian systems, splanchnic branches, and pelvic collaterals [19]. TEVAR frequently requires coverage of multiple posterior intercostal arteries, including those that supply the T8–L1 region where the artery of Adamkiewicz (AKA) most commonly arises. Evidence showed that occluding the segmental branches feeding the AKA substantially reduces ASA inflow and increases SCI risk, especially when other components of the collateral network are already compromised [20,21]. However, more recent neurosurgical and aortic literature also emphasizes that sacrifice of the AKA alone does not invariably cause infarction if the surrounding collateral network remains robust, underscoring that global network integrity may be more important than any single vessel [22,23]. In patients with long-standing aortic disease, many intercostal and lumbar arteries are already stenotic or occluded before intervention, and prior open or endovascular aortic repair (e.g., infrarenal EVAR, previous thoracic grafts) further exclude collateral reserves by eliminating additional segmental inflow levels [24]. When TEVAR is performed in this context, the sudden exclusion of remaining patent intercostal arteries may precipitate a critical reduction in perfusion pressure across the ASA and its penetrating branches. Recent meta-analyses confirm that extensive aortic coverage, thoracoabdominal involvement, and prior aortic surgery are among the strongest anatomical risk factors for SCI after endovascular repair [25,26]. Beyond simple loss of arterial inflow, several additional mechanisms contribute to SCI. First, systemic factors such as intra- or postoperative hypotension, low cardiac output, and anemia reduce spinal cord perfusion pressure, which is the difference between mean arterial pressure (MAP) and intraspinal canal pressure. Even with an anatomically intact collateral network, significant drops in MAP or increases in cerebrospinal fluid (CSF) pressure can turn the balance toward ischemia; this is the rationale for postoperative “permissive hypertension” and CSF drainage to augment spinal cord perfusion [27]. More recently, refined angiographic and CTa-based images has shown that postoperative patterns of collateralization can vary substantially between patients after segmental artery occlusion, resulting in heterogeneous perfusion and asymmetric neurologic deficits [25]. Studies of extensive TEVAR and thoracoabdominal repair also suggest that venous congestion and impaired spinal cord venous drainage may contribute to cord edema and secondary ischemia, particularly when several segmental veins are disrupted along with the arteries [28]. Taken together, these data support a model in which SCI after TEVAR emerges from the convergence of (1) loss of segmental inflow at thoracic and thoracoabdominal levels, (2) pre-existing or iatrogenic compromise of vertebral, pelvic, and paraspinal collaterals, and (3) systemic hemodynamic and microcirculatory issues that further reduce effective spinal cord perfusion. Understanding this multifactorial pathophysiology is crucial to the development of individualized prevention strategies and the rational use of adjuncts such as LSA preservation, staged repair, neuromonitoring, and CSF drainage.

#### 3.2.2. Incidence, Outcomes, and Prognosis

Although relatively uncommon, SCI is associated with major clinical consequences. Large registries and cohort studies clearly show that the occurrence of SCI after TEVAR is strongly linked to increased early and late mortality, as well as substantial long-term disability. In the National Vascular Quality Initiative (VQI) analysis of 11,473 TEVAR procedures, Scali et al. [3] reported an overall SCI rate of 3.7% (1.6% transient, 2.1% permanent); one-year survival was 87% in patients without SCI versus only 65% in those who developed SCI (54% in those with permanent deficits and 80% in those with transient deficits; *p* < 0.0001). These findings have been confirmed in more recent series of complex endovascular aortic repairs. In a 2023 multicenter study of 1681 patients undergoing branched or fenestrated EVAR, Aucoin et al. [29] reported a SCI incidence of 7.1% (3.0% permanent), with one-year survival of 90.8% in patients without SCI compared to 73.9% in those with SCI (84.8% for paraparesis vs. 66.2% for permanent paraplegia). Long-term follow-up data confirm that SCI has a sustained impact on survival and quality of life. In the landmark series by DeSart et al. [30], patients who developed SCI after TEVAR had a mean survival of 37.2 ± 4.5 months versus 71.6 ± 3.9 months for those without SCI (*p* < 0.0006); among patients with SCI, those who experienced functional improvement had a markedly better mean survival (53.9 ± 5.9 months) than those without any neurological recovery (9.6 ± 3.6 months). These data highlight not only the prognostic value of SCI itself, but also the importance of early detection and aggressive rescue strategies aimed at maximizing the chance of at least partial neurological recovery. Recent systematic reviews and meta-analyses further underscore the clinical burden of SCI in the endovascular era. Alzghari et al. [25] in an updated meta-analysis of more than 22,000 patients undergoing open or endovascular repair of descending thoracic and thoracoabdominal aneurysms, reported a pooled permanent SCI rate of 3.9% after endovascular repair, with higher rates (up to 7–8%) in extensive thoracoabdominal disease; SCI was consistently associated with significantly increased mortality across studies. More recent reports focusing on TEVAR similarly show that patients who develop SCI have dramatically worse mid-term survival—Sufali et al. [31] found 2-year survival of 69% in patients without SCI compared with only 18% in those who developed SCI after TEVAR. Importantly, even survivors with permanent or partially recovered neurological deficits experience substantial and persistent functional limitations.

#### 3.2.3. Risk Factors

The extent of aortic coverage remains the single most powerful anatomical determinant: Feezor et al. demonstrated that longer stent-graft length and more distal coverage relative to the celiac artery significantly increased SCI risk after TEVAR, with SCI rates rising from <2% in shorter segments to >8% when the thoracic coverage extended close to or beyond the celiac level [32]. These observations have been repeatedly confirmed in endovascular thoracoabdominal series, where extensive coverage of Crawford type I–III segments is associated with SCI incidences of 5–10%, particularly when no specific spinal-cord protection protocol is applied [33]. Previous aortic procedures are another major risk factor, largely because they deplete the collateral network [34,35]. Bisdas et al., in a landmark study of TEVAR, showed that patients with previous infrarenal aneurysm repair had significantly higher SCI rates (up to three-fold) than those without prior aortic surgery; preoperative renal insufficiency and prior infrarenal EVAR both emerged as independent predictors of SCI on multivariable analysis [33]. Anatomical and procedural factors relating to supra-aortic and pelvic circulation are equally important. Intentional LSA coverage without revascularization has been linked to increased SCI and posterior circulation stroke, especially when combined with dominant left vertebral arteries or bilateral hypogastric disease [36,37]. Bilateral hypogastric artery occlusion or severe iliac disease further compromises pelvic contributions to the collateral network and has been identified as an independent predictor of SCI in several fenestrated/branched EVAR (F/B-EVAR) cohorts. Emergency or acute presentations—in cases of acute complicated type B dissection or ruptured thoracoabdominal aneurysm—carry consistently higher SCI rates than elective repairs. In these settings, prolonged hypotension, limited preoperative planning, and a need for extensive aortic coverage in a single stage combine to increase risk. Recent work by Potter et al. on TEVAR for acute type B dissection with zone 3 entry tears showed that SCI, was significantly associated with longer aortic coverage and hemodynamic instability in the acute setting [38]. Similarly, the meta-analysis by Alzghari et al. found that emergency status nearly doubled the odds of SCI compared with elective repair across both open and endovascular series [25]. The presence of comorbidities, such as chronic kidney disease, diabetes, and advanced age appear to contribute additional microvascular fragility. In the large VQI-based analysis of complex endovascular repairs by Aucoin et al., chronic kidney disease and older age were independently associated not only with higher SCI incidence but also with worse post-SCI survival [29]. Doering et al. recently highlighted those intra- and early postoperative factors—such as low MAP, prolonged operative time, and need for transfusion—interact with these baseline comorbidities to shape SCI risk after F/B-EVAR [39]. In recent years, several risk prediction tools and prognostic scores have been developed to systematize this complex risk profile. Brisard et al. proposed a prognostic score for SCI during extensive endovascular repair of thoracoabdominal aneurysms that integrates aortic coverage extent, previous aortic surgery, renal function, and hypogastric patency, stratifying patients into low-, intermediate-, and high-risk categories with SCI rates ranging from about 2–3% in the lowest group to >15% in the highest [26]. Multicenter registries, such as those derived from the VQI and US Aortic Research Consortium, similarly incorporate coverage length, prior aortic repair, renal insufficiency, and emergent status to generate patient-level SCI risk estimates [29]. The major anatomical, procedural and patient-related risk factors for SCI after TEVAR are summarized in Table 1.

#### 3.2.4. Prevention Strategies

Given the multifactorial nature of SCI, prevention relies on a personalized, bundle-based approach that combines anatomical planning, staged strategies, hemodynamic optimization, and selective use of adjuncts. Preoperative planning is essential and begins with high-resolution CTa to delineate the extent of aortic coverage, identify critical segmental arteries, and assess the integrity of the collateral network, including vertebral, hypogastric and iliac vessels. This imaging-based mapping is central to the “collateral network” concept, which emphasizes that safeguarding multiple inflow sources to the spinal cord is more important than preserving any single artery in isolation [27]. LSA management plays a pivotal role within this framework. Multiple observational studies and meta-analyses have shown that not revascularized LSA coverage is associated with increased rates of both SCI and posterior stroke, particularly in patients with a dominant left vertebral artery, prior left internal mammary artery (LIMA) bypass, or contralateral vertebral disease [27]. Consequently, current practice in many high-volume centers favors selective or even routine LSA revascularization (carotid–subclavian bypass, subclavian transposition, or in situ branched stent-grafting) in patients with limited collateral reserve, extensive planned coverage, or additional risk factors (Level of evidence C) [15].

In recent years, staged repair strategies have emerged as a cornerstone of SCI prevention for extensive thoracic and thoracoabdominal pathology. Traditional staging involves performing TEVAR in two or more steps to allow time for collateral remodeling, rather than sacrificing many segmental arteries in a single step. Heidemann et al. and others have shown that staged procedures can reduce the incidence and severity of SCI compared with single-stage extensive repairs [40]. An even more refined approach is minimally invasive segmental artery coil embolization (MIS^2^ACE) or similar techniques, in which selected segmental arteries are intentionally occluded weeks before definitive aortic coverage to precondition the paraspinal collateral network. First-in-human experiences and subsequent small series suggest that this ischemic preconditioning strategy is feasible and may lower SCI risk in high-risk thoracoabdominal aneurysm patients [41]. Cerebrospinal fluid drainage (CSFD) remains a key adjunct in SCI prevention but is now used more selectively than before. According to the recent European Vascular Surgery guidelines, prophylactic CSF drainage should be considered in patients with planned extensive aortic coverage > 200 mm or previous aortic repair (level of evidence C) [15]. Data support its role in increasing spinal cord perfusion pressure (SCPP = MAP − CSF pressure), and many protocols still include prophylactic CSFD in very high-risk patients [42]. However, large registry analyses and contemporary reviews have highlighted that routine prophylactic drainage does not always decrease SCI rates and carries a small but meaningful risk of serious complications (epidural hematoma, intracranial hemorrhage, drain-related neurologic injury). Aucoin et al., using the Vascular Quality Initiative database, found that prophylactic CSFD use remained relatively stable over time, while SCI rates decreased overall with broader adoption of bundle-based prevention strategies; importantly, patients requiring therapeutic drains for established SCI had worse outcomes than those who had a prophylactic drain already in place [43]. A 2020 review by Chen et al. similarly concluded that prophylactic CSFD does not clearly reduce SCI incidence compared with no drain, while being associated with a non-negligible complication rate, advocating for individualized, selective use in patients with the highest predicted risk [1]. Recent series, including those by Rosvall et al. and Sickels et al., have incorporated CSFD as a selective component of broader SCI-prevention bundles rather than a universal procedure [44,45].

Within these bundles, hemodynamic optimization is a non-negotiable element. Most contemporary protocols target a MAP ≥ 80–90 mmHg in the immediate perioperative period, with even higher targets in the setting of new neurologic symptoms. Rosvall et al., in a 2024 study of complex F/B-EVAR, implemented a protocol that included staged repair, MAP > 80 mmHg, hemoglobin > 110 g/L, early lower-limb reperfusion, and hourly neurological checks for 36–72 h postoperatively; this approach was associated with a low incidence of persistent SCI despite extensive aortic coverage [45]. A 2025 review by Brotis et al. similarly emphasized strict blood pressure control, avoidance of hypotension, timely transfusion to maintain oxygen-carrying capacity, and rapid correction of arrhythmias or low-output states as principal components of spinal cord protection in both open and endovascular aortic surgery [42].

In complex repairs, particularly branched/fenestrated thoracoabdominal procedures, intraoperative neuromonitoring with somatosensory evoked potentials (SSEPs) and motor evoked potentials (MEPs) has become popular as a technique for early warning (level of evidence C) [15]. Although evidence remains largely observational, several high-volume centers report that intraoperative loss or significant attenuation of SSEP/MEP signals can prompt immediate interventions—raising MAP, initiating or intensifying CSFD, altering the planned extent of coverage, or accelerating completion of pelvic/limb reperfusion—Tenorio et al. and other authors highlight neuromonitoring as a valuable component of bundled strategies in extensive endovascular repairs, particularly when coupled with pre-defined, protocolized algorithms [46].

Sickels et al., in a multicenter study, showed that implementation of a standardized SCI-prevention bundle significantly reduced SCI rates after branched/fenestrated repair, with the greatest benefit observed in high-risk patients [44]. A recent systematic review by Lella et al. further supports the concept that SCI mitigation is most successful when multiple complementary measures—anatomical planning, staging, MAP optimization, CSFD, LSA/pelvic preservation, and neuromonitoring—are applied in a coordinated, patient-tailored fashion rather than in isolation [27].

More recently, artificial intelligence (AI)–based approaches have been proposed as emerging tools to improve preoperative risk stratification for spinal cord ischemia (SCI) following TEVAR. Machine-learning models combining preoperative clinical parameters, procedural details, and advanced imaging data—particularly computed tomography angiography (CTA)—have been developed to estimate individual SCI risk [47]. These models rely on automated extraction of vascular and clinical features and may assist in identifying patients who could benefit from intensified preventive strategies, such as staged repair, selective cerebrospinal fluid drainage, or tailored hemodynamic management. In parallel, multimodal AI platforms integrating clinical data, imaging, biomarkers, and physiologic parameters (e.g., projects such as VASCUL-AID) aim to predict aortic disease progression and the likelihood of major complications, including SCI [48]. Deep-learning techniques for automated segmentation of the aortic tree and segmental arteries further enable objective assessment of collateral networks, potentially improving anatomical planning and intraoperative risk estimation. In addition, in silico and data-driven computational models have been introduced to simulate post-repair spinal cord perfusion and to estimate the hemodynamic impact of segmental artery coverage, representing a promising AI-enabled adjunct to clinical decision support [49]. Although current evidence remains limited and largely exploratory, these approaches highlight the potential role of AI in advancing personalized SCI prevention strategies and warrant prospective validation in dedicated TEVAR and thoracoabdominal endovascular repair cohorts.

#### 3.2.5. Management of Established Spinal Cord Ischemia (SCI)

When spinal cord ischemia becomes clinically apparent, prompt and aggressive intervention is critical. In most centers, recognition of new lower-limb weakness, sensory change, or sphincter dysfunction after TEVAR triggers a standardized “rescue” protocol. The first step is a rapid clinical assessment (including sedation hold, focused neurological examination, and exclusion of alternative causes such as epidural hematoma), followed immediately by escalation of spinal cord–protective measures. Increasing mean arterial pressure (MAP)—typically to ≥90–100 mmHg using vasopressors—is a universal cornerstone, as spinal cord perfusion pressure depends on the gradient between MAP and cerebrospinal fluid (CSF) pressure [42].

CSF drainage plays a central role in rescue therapy. If a drain is already in place, most protocols lower the CSF pressure target from around 10 mmHg to 5–8 mmHg to increase spinal cord perfusion pressure; if no drain is present, urgent therapeutic lumbar CSF drainage is instituted whenever not contraindicated [50]. Concomitantly, hemoglobin is optimized (commonly to ≥9–10 g/dL), oxygenation and ventilation are corrected, and any hypotension, arrhythmias, or low-output states are aggressively treated. Reversible anatomical causes—such as acute LSA occlusion after zone 2 TEVAR, extensive iliac thrombosis, or critical hypogastric compromise—are actively sought with urgent imaging and, when identified, addressed with secondary interventions (e.g., LSA revascularization, iliac or hypogastric re-opening) [28,42].

Recent data support the effectiveness of such bundled rescue therapy. In a large single-center series of 943 TEVAR/fenestrated–branched procedures, Spratt et al. reported post-TEVAR SCI after 7.8% of procedures; among patients treated with a standardized rescue protocol (MAP ≥ 100 mmHg, urgent CSF drainage or intensification, hemoglobin 9–10 g/dL, LSA revascularization as needed, plus pharmacologic adjuncts), partial or complete neurological improvement occurred in 68.9% of SCI episodes, and complete recovery in ~50%, with permanent paraplegia limited to about 1% of the overall cohort [51]. The same group showed that CSF drainage had the strongest association with neurologic improvement among rescue interventions, although it carries a non-negligible risk of complications and thus must be managed in experienced hands.

As reported by the 2024 ESC Guidelines for Peripheral Arterial and Aortic Diseases and the 2024 ESVS Clinical Practice Guidelines for abdominal aorto-iliac aneurysms [52,53], new neurological deficit after thoracic or thoracoabdominal aortic repair should trigger immediate intensification of spinal cord protection: rapid MAP augmentation, urgent consideration of CSF drainage in suitable patients, correction of anemia and hypoxia, and assessment for correctable anatomical defects. Although outcomes remain heterogeneous and some patients progress to permanent deficits despite optimal therapy, these contemporary data demonstrate that early recognition and protocolized rescue treatment can meaningfully improve neurological outcomes in a substantial proportion of patients with post-TEVAR SCI. This underlines the importance of rigorous postoperative neurological surveillance, clear institutional algorithms, and close collaboration among vascular surgeons, anesthesiologists, and neurology/neurosurgery teams.

### 3.3. Delirium and Cognitive Outcomes

Postoperative delirium is an increasingly recognized complication of TEVAR, particularly among elderly patients and those undergoing urgent procedures for complicated type B dissection. In a cohort of 434 patients with complicated type B aortic dissection treated with TEVAR, Liu et al. reported a postoperative delirium (POD) incidence of 13.3%, with delirium independently associated with longer Intensive care unit (ICU) stay, prolonged hospitalization, and higher early adverse event rates. Older age, preoperative hyponatremia, elevated creatinine, and prolonged mechanical ventilation emerged as significant predictors in multivariable analysis [54]. Huang et al. [55] developed and validated a TEVAR-specific nomogram for POD in type B dissection patients, incorporating variables such as age, renal function, serum sodium, and intraoperative factors; the model supports its use for individualized risk stratification and targeted prevention.

Beyond the TEVAR setting, a broader cardiovascular and aortic surgery literature confirms the clinical weight of delirium. Meta-analyses and large cohort studies in acute type A aortic dissection and complex cardiac surgery consistently report POD incidences ranging from 20% to over 50%, with delirium associated with higher in-hospital mortality, increased institutionalization, and greater risk of incident dementia and long-term cognitive decline [56]. Recent prediction models for delirium in cardiovascular surgery (e.g., Xu et al., 2022; Liu et al., 2024; Mei et al., 2025) highlight a recurring set of risk factors—advanced age, pre-existing cognitive impairment, renal dysfunction, low albumin, benzodiazepine use, poor preoperative sleep quality, and prolonged cardiopulmonary bypass or operative time—that mirror many of the determinants identified in the TEVAR/type B dissection population [57,58,59].

More broadly, pre-existing cognitive impairment is now recognized as a key substrate for both delirium and postoperative cognitive decline. In a systematic review and meta-analysis, Houghton et al. estimated that approximately 61% of patients with vascular pathology had evidence of cognitive impairment before surgery [8]. Goodijk et al. confirmed that almost 50% of vascular surgery patients had a preoperative cognitive deficit and that worse baseline cognitive performance is associated with higher rates of postoperative complications and delirium, as well as greater functional decline [60]. In a separate cohort, Styra et al. showed that moderate-to-severe cognitive impairment, according to Montreal Cognitive Assessment (MoCA) ≤ 15, increased the odds of delirium more than six-fold after major vascular procedures, and that open aortic surgery—compared with less invasive operations—carried a particularly high delirium risk [61].

These data reinforce the rationale for personalized perioperative strategies aimed at reducing delirium and preserving cognitive function in TEVAR candidates. Practical components of such an approach include:

- Preoperative cognitive screening (e.g., MoCA or other validated tools) to identify high-risk patients who may benefit from closer monitoring and targeted prevention.
- Optimization of modifiable risk factors, such as correction of hyponatremia and other electrolyte disturbances, meticulous management of renal function, and avoidance of unnecessary benzodiazepines and anticholinergic agents [54].
- Implementation of a multimodal delirium-prevention bundle, comprising orientation aids, preservation of circadian rhythm and sleep, early mobilization, adequate pain control with opioid-sparing strategies.

Postoperative delirium and neurocognitive changes after TEVAR are not inevitable factors of high-risk surgery, but rather expressions of an interaction between procedural stressors and individual vulnerability.

### 3.4. Other Neurological Complications

Although less common than SCI or stroke, several additional neurological complications may occur after TEVAR and deserve consideration because they can significantly affect recovery and quality of life. Among those, seizures secondary to cerebral ischemia, posterior reversible encephalopathy syndrome (PRES), or metabolic disturbances may occur. In most TEVAR series they are reported only sporadically and often in association with overt stroke or encephalopathy rather than as isolated events [62]. Case reports and small series of complex endovascular aortic repair (including thoracoabdominal and arch procedures) describe generalized tonic–clonic seizures as a presenting feature of PRES or diffuse cerebral edema, typically occurring within the first 24–72 h postoperatively and often accompanied by visual disturbances or altered mental status [63,64].

Posterior reversible encephalopathy syndrome (PRES) represents a rare but increasingly recognized cause of post-TEVAR encephalopathy. PRES is characterized by headache, seizures, visual symptoms, and altered consciousness, with vasogenic edema predominantly in the parieto-occipital lobes on magnetic resonance images MRI [65]. Although evidence comes from oncology, obstetrics, and general critical care patients, there are case reports describing PRES after complex endovascular aortic repair, typically in the setting of marked blood pressure fluctuations or aggressive antihypertensive therapy [63,66]. In these series, most patients recover fully or almost fully with prompt blood pressure control, withdrawal of precipitating agents, and antiepileptic therapy, but up to 10–15% may have incomplete radiological or clinical resolution, especially when diagnosis or treatment is delayed [65].

Peripheral nerve injury is another infrequent but important complication, most often related to large-bore access or positioning rather than to the endograft itself. Thoracic endografts typically require large femoral sheaths, which increase the risk of access-site bleeding, pseudoaneurysm, or retroperitoneal hematoma—each of which can compress adjacent neural structures. Reviews of angiography and endovascular interventions estimate that femoral neuropathy occurs in approximately 0.2% of patients undergoing femoral arterial access, usually in association with a large groin or iliopsoas hematoma or pseudoaneurysm [67]. In broader cardiovascular series, pseudoaneurysm is reported as the most common femoral access complication, with an incidence ranging from 0.2% to 8% depending on sheath size, antithrombotic regimen, and puncture technique. While dedicated TEVAR access studies rarely report precise rates of nerve injury, upper- and lower-extremity access series in F/B EVAR confirm that clinically evident peripheral neuropathy is rare but can lead to prolonged pain, weakness, or sensory loss when it occurs [68,69]. These observations reinforce the importance of ultrasound-guided puncture, meticulous hemostasis, and early recognition of expanding hematomas or pseudoaneurysms in TEVAR patients.

Cholesterol embolization syndrome (CES) represents another rare but notable neurologic and systemic complication after TEVAR. CES results from embolization of cholesterol crystals from ulcerated aortic plaques, leading to multifocal occlusion of small arteries with combined ischemic and inflammatory injury [70]. In large vascular and cardiac catheterization series, the incidence of clinically evident CES ranges from 0.09% to 2.9%, although autopsy studies suggest much higher rates (up to 12–77%) in selected populations, such as elderly patients dying after aortic surgery or aortography, indicating that many cases remain subclinical or unrecognized [70]. CES typically presents with a combination of renal dysfunction, livedo reticularis, digital ischemia, and eosinophilia, but cerebral and retinal embolization can also occur. Case reports describe multifocal cerebral infarctions due to CES following TEVAR in patients with “shaggy” thoracic aortas, sometimes despite technically successful stent-graft deployment; in one recent report, a 75-year-old man developed multiple cortical infarcts shortly after zone 2 TEVAR with LSA embolization, with imaging and clinical features consistent with cholesterol crystal embolism rather than thromboembolism alone [71].

Overall, these “non-core” neurological complications—seizures (often in the context of stroke or PRES), access-related peripheral neuropathies, and cholesterol embolization syndromes—are individually rare but collectively important. They broaden the spectrum of neurologic vulnerability after TEVAR and highlight the need for comprehensive surveillance that extends beyond overt SCI and stroke.

## 4. Gaps in Knowledge and Future Directions

Despite remarkable advancements, there are still substantial gaps in our knowledge of and ability to prevent neurological complications following TEVAR. To increase comparability among research, standardized definitions and reporting requirements for neurological events—such as small strokes, temporary SCI, and cognitive findings—are crucial. There are few prospective studies assessing neuroprotective strategies, especially the function of selective vs. routine CSFD, which is an important field for further investigation. Similarly, SCI and delirium risk scores need to be externally validated and refined, maybe using biomarkers, new imaging modalities, or machine learning-based techniques. Lastly, there is an increasing demand for long-term neurocognitive outcome data specific to TEVAR patients, such as studies utilizing neuropsychological evaluation and brain MRI.

This review has several limitations that should be acknowledged. First, the narrative and non-systematic design exposes the review to potential selection bias, as study inclusion was based on relevance and methodological quality rather than on predefined systematic criteria. Consequently, no formal risk-of-bias assessment or quantitative meta-analysis was performed, and the relative weight of individual studies cannot be statistically compared. Second, the available literature on neurological complications after TEVAR is highly heterogeneous with respect to patient populations, aortic pathology, procedural complexity, definitions of neurological outcomes (particularly transient versus permanent spinal cord ischemia), and follow-up duration, limiting direct comparability across studies. Third, most evidence derives from retrospective cohorts and large registries, while prospective randomized data—especially on preventive strategies such as cerebrospinal fluid drainage, neuromonitoring, or staged repair—remain scarce. In addition, emerging topics discussed in this review, including artificial intelligence–based risk prediction and decision-support tools, are currently supported by preliminary and largely exploratory studies, with limited external validation and no prospective implementation trials specific to spinal cord ischemia after TEVAR. Finally, long-term neurocognitive outcomes, patient-reported functional status, and quality-of-life measures remain underreported in the existing literature, representing an important need for future research.

## 5. Conclusions

Following TEVAR, neurological complications—particularly SCI, stroke, and delirium—remain uncommon but extremely significant events that impact affected patients’ long-term quality of life and survival. Large registries, systematic reviews, and evidence-based studies have highlighted important risk variables during the past 20 years and provided guidance for a more personalized approach to care and prevention. The best current method for reducing neurologic damage while preserving the benefits of endovascular repair is a customized approach that incorporates anatomical complexity, previous aortic interventions, comorbidities, and cognitive vulnerability, while selectively utilizing tools like LSA revascularization, staged repair, CSFD, neuromonitoring, and tailored hemodynamic targets.

## Figures and Tables

**Table 1:** Summary of the major risk factor for spinal cord ischemia (SCI).

Risk Factor	Effect on SCI Risk	Evidence
Long extent of aortic coverage	Strongly increases up to >8–10%	Feezor et al. [32]; Bisdas et al. [33].
Previous aortic surgery	Up to three-fold increase	Bisdas et al. [33].
LSA coverage without revascularization	Increased risk of SCI and posterior stroke	Hiraoka et al. [37]; Xodo et al. [36].
Bilateral hypogastric artery occlusion	Independent predictor of SCI	Xodo et al. [36].
Acute complicated type B dissection	Nearly double SCI risk	Potter et al. [38]; Alzghari et al. [25].
Intra or post-operative low mean arterial pressure	Reduce spinal cord perfusion pressure	Doering et al. [39].
Prolonged operative time	Associated with higher SCI incidence	Doering et al. [39]; Bisdas et al. [33].
Extensive single-stage repair	Associated with higher SCI incidence	Doering et al. [39].
Chronic kidney disease	Independent predictor of SCI	Aucoin et al [29].
Combined assessment of coverage length, prior surgery, renal insufficiency	Stratifies SCI risk from 2–3% up to 15%	Brisard et al [26].

SCI: Spinal cord ischemia; LSA: Left subclavian artery.

## Data Availability

No new data were created or analyzed in this study. Data sharing is not applicable to this article.

## Abbreviations

The following abbreviations are used in this manuscript:

TEVAR	Thoracic Endovascular Aortic Repair
SCI	Spinal Cord Ischemia
LSA	Left Subclavian Artery
CSF	Cerebro Spinal Fluid
CTa	Computed Tomography Angiography
ASA	Anterior Spinal Artery
AKA	Adamkiewicz Artery
EVAR	Endovascular Aortic Repair
MAP	Mean Arterial Pressure
VQI	Vascular Quality Initiative
LIMA	Left Internal Mammary Artery
MIS^2^ACE	Minimally Invasive Segmental Artery Coil Embolization
CSFD	Cerebro Spinal Fluid Drainage
SCPP	Spinal Cord Perfusion Pressure
F/B EVAR	Fenestrated/Branch Endovascular Aortic Repair
SSEPs	Somatosensory Evoked Potentials
MEPs	Motor Evoked Potentials
POD	Postoperative Delirium
ICU	Intensive Care Unit
MoCA	Montreal Cognitive Assessment
PRES	Posterior Reversible Encephalopathy Syndrome
MRI	Magnetic Resonance Imaging
CES	Cholesterol Embolization Syndrome
